# The impact of chelation compliance in health outcome and health related quality of life in thalassaemia patients: a systematic review

**DOI:** 10.1186/s12955-023-02221-y

**Published:** 2024-02-02

**Authors:** Wan Jin Lee, Nurul Ain Mohd Tahir, Geok Ying Chun, Shu Chuen Li

**Affiliations:** 1 Centre of Quality Management of Medicines, Faculty of Pharmacy, Universiti Kebangsaan Malaysia, Kuala Lumpur, Malaysia; 2 School of Biomedical Sciences and Pharmacy, College of Health, Medicine and Wellbeing, University of Newcastle, New South Wales, Australia

**Keywords:** Compliance, Iron chelation therapies, Thalassaemia, HRQoL, Iron overload

## Abstract

Understanding consequences of poor chelation compliance is crucial given the enormous burden of post-transfusional iron overload complications. We systematically reviewed iron-chelation therapy (ICT) compliance, and the relationship between compliance with health outcome and health-related quality of life (HRQoL) in thalassaemia patients. Several reviewers performed systematic search strategy of literature through PubMed, Scopus, and EBSCOhost. The preferred reporting items of systematic reviews and meta-analyses (PRISMA) guidelines were followed. Of 4917 studies, 20 publications were included. The ICT compliance rate ranges from 20.93 to 75.3%. It also varied per agent, ranging from 48.84 to 85.1% for desferioxamine, 87.2–92.2% for deferiprone and 90–100% for deferasirox. Majority of studies (N = 10/11, 90.91%) demonstrated significantly negative correlation between compliance and serum ferritin, while numerous studies revealed poor ICT compliance linked with increased risk of liver disease (N = 4/7, 57.14%) and cardiac disease (N = 6/8, 75%), endocrinologic morbidity (N = 4/5, 90%), and lower HRQoL (N = 4/6, 66.67%). Inadequate compliance to ICT therapy is common. Higher compliance is correlated with lower serum ferritin, lower risk of complications, and higher HRQoL. These findings should be interpreted with caution given the few numbers of evidence.

## Introduction

Thalassaemia is a hereditary disorder, characterised by abnormal globin chain synthesis in the haemoglobin molecules, affecting millions of people around the world and resulting in thousands of deaths annually [1]. Around 1.5% of the world’s population was found to be carriers of beta-thalassaemia [2]; with the prevalence estimated to range between 0.16 and 25 per 100,000 population in Europe, 4 per 100,000 population in North Africa, and 11–36 per 100,000 population in the Middle East [3].

Thalassaemia poses a high financial burden and a huge psychological burden on families, societies, and healthcare systems. Clinically, chronic blood transfusion is required for thalassaemia patients to attenuate anaemia and increase the haemoglobin level. However, multiple episodes of blood transfusion to achieve these goals will have an effect on serum ferritin levels. Systemic iron overload can lead to iron accumulation in the heart, liver, spleen, and other organs, which can cause a variety of complications. The risk of serious iron overload complications is an alarming clinical concern, and properly utilized iron chelation therapy (ICT) is crucial to manage post-transfusional iron overload.

To date, there are three iron chelators available in the market: desferrioxamine (DFO), deferiprone (DFP), and deferasirox (DFX). DFO is the first-line iron chelation treatment for children and adults in Malaysia [4]. Ophthalmic, auditory, and bone abnormalities, growth retardation, skin allergies are recognized as the side effects related to DFO. Furthermore, at higher doses, neurological, and pulmonary issue have been observed [5]. Physician might switch the iron chelator to DFP or DFX or their combination of therapy in the cases of contraindication or side effects and inadequate chelation. Agranulocytosis, and gastrointestinal issues due to DFP [6] are common in TDT patients. The neutrophil count is recommended to be assessed weekly and 10% of patients permanently discontinue DFP due to its side effects [6, 7]. Meanwhile, patents prescribed with DFX commonly experience gastrointestinal effects, a rise in serum creatinine, and skin rashes [8]. Adjustments to the iron chelator regimen are necessary in response to adverse effects or when the desired serum ferritin levels are not achieved.

However, non-compliance with iron chelators persists as a major and enduring issue in transfusion-dependent thalassaemia patients. The most common obstacle in enhancing chelation compliance is patient-related, such as a lack of psychological willingness and belief in their ability in administering ICT, which is often due to failure in regulating their negative emotions [9]. The estimated mean rate of patients’ compliance of desferrioxamine (DFO) is often dissatisfactory, ranging from 59 to 78% [10]. Low compliance to iron chelation therapy jeopardises treatment effectiveness, resulting in significant morbidity and mortality, reducing patients’ health-related quality of life, as well as higher expenses to manage iron overload complications.

Many studies on thalassaemia and compliance to iron chelation have been conducted in a limited context around the world. Previous studies [11, 12] systematically reviewed chelation adherence in thalassemia patients, while another study by Arian et al. (2019) [13] reviewed the health-related quality of life associated with thalassaemia patients but did not measure the ICT compliance among them. A review by Delea et al. (2007) [10] investigated the rate of compliance of desferrioxamine (DFO) and deferiprone (DFP) only without deferasirox (DFX) and the association between compliance and iron overload complications, focusing on health outcomes such as cardiac diseases, and diabetes using a small number of articles (as limited relevant studies were conducted at that time) [10]. Hence, there still exists some deficit in our knowledge on the impact of ICT therapy compliance on various clinical and patient-relevant outcomes in patients with thalassaemia. The objective of our current study was to further fill the gaps in measuring compliance rate in all ICT (DFO, DFP & DFX and the combination therapy), understanding the impact of chelation compliance on clinical outcomes and health-related quality of life in all populations of thalassaemia patients.

## Methods

This systematic review was conducted following the Preferred Reporting Items for Systematic Reviews and Meta-Analysis statement (PRISMA) to achieve high-quality and transparent research reporting [14].

### Search terms

Relevant articles were searched through PubMed, Scopus, and EBSCOhost from the inception of these databases until 1st February 2022. The medical subject headings (MeSH) terms and keywords used were “medication adherence” OR “medication compliance” OR “treatment adherence” OR “treatment compliance” OR “Treatment Adherence and Compliance“[Mesh] AND “quality of life” OR “SF36” OR “EQ-5D” or “health-related quality of life” OR “survival rate” OR “quality adjusted life years” or “Quality of Life“[Mesh] AND “health outcome*” OR “health result*” OR “medical outcome*” OR “medical result*” OR “MRI” OR “Serum ferritin” OR “Iron overload complication*” OR “iron related complication*” OR “survival rate” OR “Quality adjusted life years” OR “Outcome Assessment, Health Care“[Mesh] OR “clinical outcome*” OR “clinical result*” OR “healthcare outcome*” OR “healthcare result*” OR “medical care outcome*” OR “medical care result” OR “health related outcome*” OR “health related result*” OR “medical related outcome*” OR “medical related result*” AND “Thalassemia” OR “thalassaemia” OR “beta thalassemia” OR “beta thalassaemia” OR “beta thalassemia” [Mesh].

### Inclusion and exclusion criteria

The selection of publications was based on the population, intervention, comparison, outcome, and study (PICOS) approach.

- Population: Thalassaemia patients were prescribed iron chelation therapy (ICT). ICT utilisation in patients with comorbidities of sickle cell disease (SCD), myelodysplastic syndrome (MDS) and other diseases was excluded.
- Types of interventions: Monotherapy or combination of iron chelation therapy such as desferrioxamine (DFO), deferiprone (DFP), deferasirox (DFX), DFO& DFP, DFO & DFX and DFX & DFP.
- Types of outcome measures: The compliance outcomes in terms of the rates or percentage. Health outcomes include serum ferritin levels, MRI T2*, iron overload complications and health-related quality of life (HRQoL). The outcomes must be reported as comparisons between the compliant and non-compliant groups. Articles focusing on determinants of chelation compliance were not included.
- Study design: Study designs included in this review were original clinical studies, for example, cross-sectional studies, cohort studies and randomised controlled trials were included. Only full-text original articles in English were included. Editorials, expert opinions, conference abstracts, case studies or series, study protocols or reviews were excluded.

### Study selection

The title and abstract of the articles were independently evaluated based on the inclusion and exclusion criteria by two investigators (WJ and GY). Subsequently, the full text targeted articles were retrieved and accessed for eligibility to be included. The reason for the articles’ exclusion was documented. The process of study selection, data extraction and quality appraisal were conducted independently by two investigators (WJ and GY). Any disagreements or differences in opinion between the two researchers (WJ and GY) were handled through discussions and consensus, followed by a third researcher’s independent opinion (NAMT). The three researchers (WJ, GY and NAMT) would need to reach an agreement before making a final decision.

### Data extraction

Variables assessed included the study’s characteristics, such as the year of publication, the country where the study was conducted, sample size, study design, and study duration through a standardized data collection form. The compliance outcomes in terms of rates and/or percentages were extracted from the included articles. Health outcome measures such as serum ferritin levels, cardiac and iron loading, endocrine related complications, and health-related quality of life were collected and tabulated accordingly.

### Quality appraisal

The risk of bias and the methodology quality of the identified publications were assessed using the Newcastle-Ottawa Scale (NOS) for cohort studies and a NOS adapted version for cross-sectional studies [15]. NOS is a star-rating based system, with a maximum of 9 scores for cohort studies, randomized controlled trials and a maximum of 10 scores for cross-sectional studies. Each study’s quality is assessed using the following grading algorithms: with a NOS score of 7 and above, it is considered a high-quality study, studies with 4 to 6 points are considered medium-quality, while those with 0 to 3 scores are considered low-quality [16].

## Results

### Literature selection

**Fig. 1:**
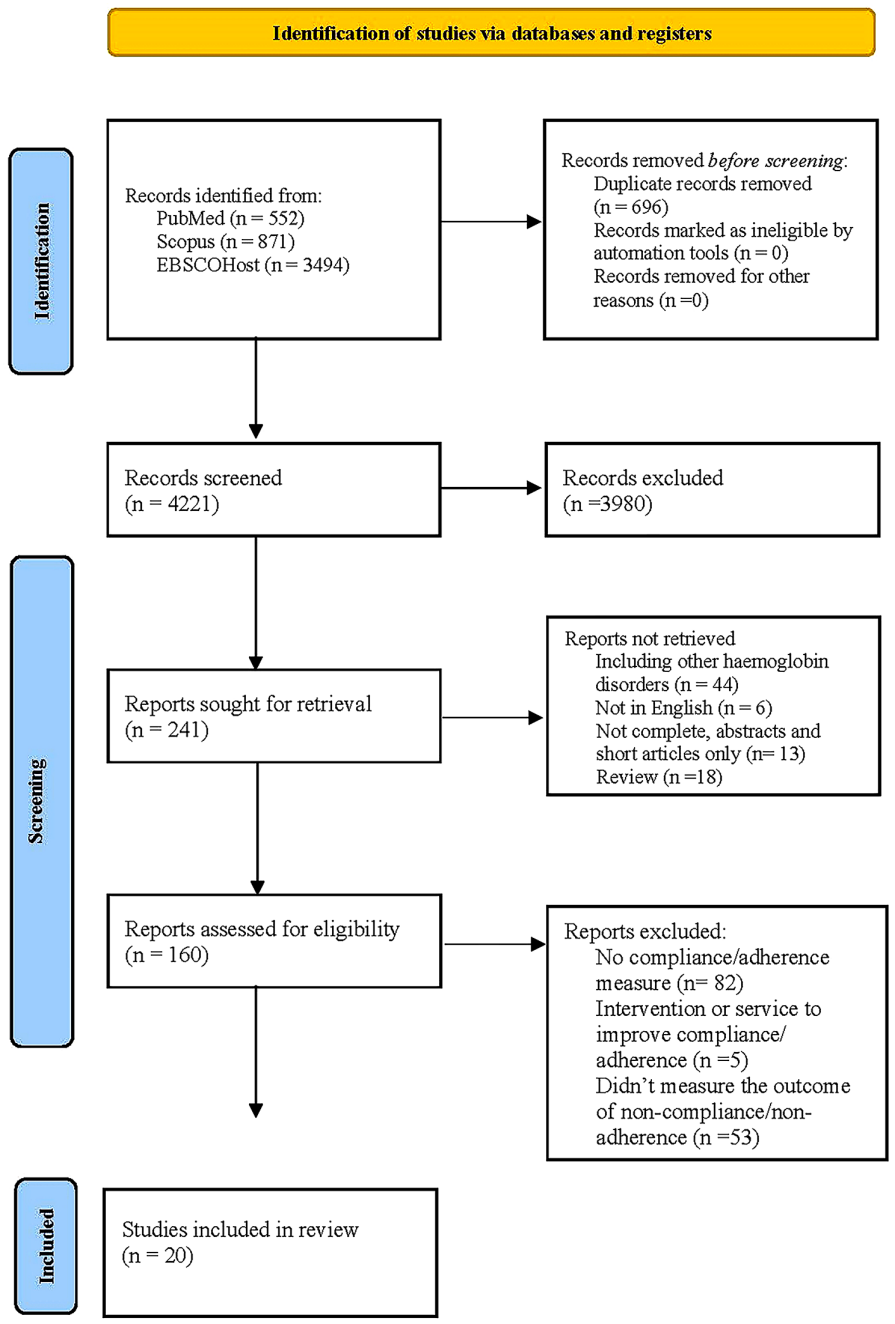
PRISMA 2020 Flow Diagram on the literature selection process for a systematic review of the impact of chelation compliance on health outcomes and health-related quality of life on thalassemia patients

A total of 4917 studies was identified from the selected databases of PubMed, Scopus and EBSCOHost and 696 studies were eliminated due to duplication of titles. The remaining 4221 articles were screened, and 3980 articles were excluded because of irrelevant titles. Meanwhile, 44 articles involving other haemoglobin disorders such as sickle cell disease (SCD) and myelodysplastic syndrome (MDS), 6 non-English articles, 13 abstract-only articles, and 18 review articles were removed after assessing the abstracts. The eligibility of the remaining 160 full-text articles was evaluated. In this process, 82 publications without adherence/compliance measures, 5 studies focused on intervention or healthcare providers’ services, and 53 articles that only evaluated the determinants of chelation measure without comparing the outcomes among the compliant and non-compliant population were removed. Finally, only 20 articles were eligible for synthesis after the culling process. The summary of the literature selection process in this review is shown in Fig. 1.

### Study characteristics

The characteristics of the studies included in the research are tabulated in Table 1. The published articles were of worldwide origins and were conducted in countries including the United Kingdom, Italy, the United States, Canada, Australia, Singapore, Malaysia, Thailand, Iran, Egypt, Syria, and India. Most of the studies were cross-sectional studies (N = 16, 80%) [17–32], followed by cohort studies (N = 3, 15%) [33–35], and only one randomised controlled trial [36]. The majority of the studies were single-centred (N = 13, 65%) and about 30% of the studies were conducted in multiple settings [18, 23, 30, 31, 33, 36], while the remaining study did not mention the setting [17].

A variety of medication measures was used across the studies. Most of the studies (N = 7, 35%) examined the rate of compliance using the frequency of ICT’s administration [17, 18, 20, 22, 24, 25, 29], followed by studies that evaluated patients’ compliance status using vial or pill count (N = 5, 25%) [19, 33–36]. Several studies (N = 3, 15%) used the Likert Scale [23, 27, 32], while some studies (N = 3, 15%) measured compliance using self-reported questionnaires such as Morisky Medication Adherence Scale (MMAS-8) [28], the Medication Compliance Questionnaire (MCQ) [30], and standardised questionnaire (not mentioned in the study) [31]. Meanwhile, a study [26] reported the compliance with medication possession ratio (MPR), and another study [21] did not mention the method of compliance measurement.

**Table 1:** Study characteristics of published literature on the impact of compliance on health outcome and health-related quality of life on thalassaemia patients

Author, Year	Country	Sample size	Centre	Study Design	Iron Chelation Therapy	Duration
Wolfe et al. 1985 [33]	United States	36	Multi	Prospective cohort	DFO	6 years
Al-Refaie et al. 1992 [17]	United Kingdom	52	N/A	Cross sectional	DFO	N/A
Richardson et al. 1993 [34]	Australia	76	Single	Retrospective cohort	DFO	N/A
Arboretti et al. 2001 [18]	Italy	867	Multi	Cross sectional	DFO	Period 1: 5 months Period 2: 6 months
Kidson-Gerber et al. 2008 [19]	Australia	44	Single	Phase 1: Cross sectional Phase 2: Retrospective	DFO (n = 43), DFP (n = 18), DFO & DFP (n = 17)	1 year
Lee et al. 2011 [20]	Malaysia	139	Single	Cross sectional	DFO	1 year
Haghpanah et al. 2013 [21]	Iran	101	Single	Cross sectional	DFO	N/A (2009)
Mokhtar et al. 2013 [35]	Egypt	447	Single	Retrospective cohort	DFO (n = 99), DFP (n = 119), DFX (n = 21) and DFO& DFP (n = 208)	N/A
Haghpanah et al. 2014 [22]	Iran	220	Single	Cross sectional	DFO (n = 114), DFX (n = 106)	N/A (2012)
Elalfy et al. 2014 [36]	Egypt	96	Multi	Prospective randomized controlled trial	Group A: DFO & DFP (n = 48) Group B: DFP & DFX (n = 48)	1 year
Sobota et al. 2014 [23]	US, Canada, and the UK	264	Multi	Cross sectional	DFO (n = 57), DFP (n = 136), DFX (n = 9), DFO & DFP (n = 21) and DFO & DFX (n = 17), patients with no chelation (n = 22) Patients on no chelation were excluded from some analyses that focused on chelator choice.	N/A
Bazi et al. 2017 [24]	Iran	80	Single	Cross sectional	N/A Monotherapy (n = 62) and combinational regimen (n = 18)	N/A
Sobhani et al. 2019 [25]	Iran	90	Single	Cross sectional	DFO (n = 52), DFX (n = 29), DFO & DFX (n = 9)	1 year
Yassouf et al. 2019 [26]	Syria	82	Single	Cross sectional	DFO	3 months
Sukhmani et al. 2020 [27]	India	215	Single	Cross sectional	DFO, DFP, DFX and combination therapy 57.2% of patients (n = 123) were on monotherapy and 42.7% of patients (n = 92) on combinational therapy of two or more iron chelators.	N/A
Theppornpitak et al. 2021 [28]	Thailand	70	Single	Prospective cross sectional	DFP (n = 49), DFX (n = 14), DFO (n = 2)	5 months
Badur et al. 2021 [32]	Turkey	27	Single	Prospective cross sectional	N/A	N/A
Mahmoud et al. 2021 [29]	Egypt	120	Single	Cross sectional	DFX, DFO or both	2 years
Chai et al. 2021 [30]	Malaysia	198	Multi	Prospective cross sectional	DFO (n = 32), DFP (n = 60), DFX (n = 73), DFO & DFP (n = 20), DFO & DFX (n = 13)	1 year
Lam et al. 2021 [31]	Singapore	73	Multi	Ambidirectional cross sectional study Retrospective reviews of clinical information and prospective interview patients/caregivers for socioeconomic data and compliance	38.2% of subjects were on monotherapy with DFX, 17.6% on DFP, 8.8% on DFO and 35.3% were on combination therapy.	2.5 years

Abbreviations and Footnotes: DFO = desferrioxamine ; DFP = deferiprone; DFX = deferasirox; N/A = not available

### Quality assessment

The details of the methodological quality assessment of the studies are tabulated and summarised in Table 2. To sum up, more than half of the studies (N = 12, 60%) were considered high-quality studies, while the remaining studies (N = 8, 40%) were considered medium-quality studies.

### Newcastle-Ottawa scale for cross-sectional study (N = 16)

Overall, most of the cross-sectional studies (N = 11) clearly defined the representative sample of the thalassaemia population, but there were no descriptions of the population in 5 of the studies [17, 18, 26, 29, 32]. Only 5 studies reported having a sufficient sample size following an appropriate formula of sample size estimation [18–20, 25, 30].

One study had an unsatisfactory response rate (less than 80%) [31] while there were no descriptions of the response rate nor the characteristics of the respondents and the non-respondents in 4 studies [20, 22, 28, 32].

All of the studies measured compliance rates using validated measurement tools or were able to describe the measurement tool except in the study by Haghpanah et al. (2013) [21].

Aside from that, 6 studies were not adequately designed or analysed to control confounders with regard to demographic factors such as education level, age, income, and treatment type [17, 19, 25–28]. For the remaining 10 studies, the comparability among the different outcome groups was shown and the confounding factors were controlled. In these studies, recruited samples were matched in age, gender or other significant factors [18, 22, 23, 25]; data analysed in multiple logistic regression while controlling multiple factors such as income levels, and family history that would affect compliance [20, 21, 29, 30]; or the outcome stratified based on gender [32], sociodemographic and clinical factors [31]. The data in all the 16 cross-sectional studies were assessed from reliable resources such as medical records, laboratory investigation, and validated questionnaires. They also used appropriate statistical tests and described the measurement of the relationship among outcomes of interest.

### Newcastle-Ottawa scale for cohort study (N = 3)

All the cohort studies [33–35] had representative samples of the thalassaemia population and their non-exposed cohort were drawn from the same population as the exposed cohort. During the selection of the cohort study, only Wolfe et al. (1985) [33] reported that all the patients did not have a cardiac disease (outcome of interest). The comparability of cohorts on the basis of the design or analysis was appropriate and justifiable. Two studies recruited both arms from a similar sociodemographic background, while the other study [34] used logistic regression to explain the relationship between multiple variables and the outcome of interest. All the studies assessed the outcome through independent blind assessment. All studies had an adequate period for assessing the outcome ranging between 6 years to 12 years and complete follow-up of the subjects. Wolfe et al. (1985) [33] prospectively followed up the patients for 6 years without any dropouts, while the other studies retrospectively review patients attended the clinic for 12 years [34] and 10 years [35].

### Newcastle-Ottawa scale for randomized controlled trial (N = 1)

The sole randomized controlled trial in this study is considered to have high quality [36]. The participants of the study were regular thalassaemia attendees of the Thalassaemia Centers, Ain Shams University, Egypt and Sultan Qaboos University Hospital Oman who had severe iron overload defined as serum ferritin > 2500ng/mL, liver iron concentration > 7 mg/g and cardiac T2* <20 and > 6 ms without heart dysfunction. The case and control groups were adequately defined and appropriately represented the targeted population. The cases and controls were age and gender matched. The data were then assessed through laboratory test and SF-36 health survey for both groups without dropouts.

**Table 2:** Methodological assessment of the studies through Newcastle Ottawa scale

Cross-Sectional Studies									
Study	Representativeness	Sample size	Non-respondents	Ascertainment of exposure	Comparability	Assessment of outcome	Statistical test		Score
Al-Refaie et al. 1992 [17]			*	*		**	*		5
Arboretti et al. 2001 [18]		*	*	*	*	**	*		7
Kidson-Gerber et al. 2006 [19]	*	*	*	*		**	*		7
Lee et al. 2011 [20]	*	*		**	*	**	*		8
Haghpanah et al. 2013 [21]	*		*		*	**	*		6
Haghpanah et al. 2014 [22]	*			*	*	**	*		6
Sobota et al. 2014 [23]	*		*	*	**	**	*		8
Bazi et al. 2017 [24]	*		*	*	*	**	*		7
Sobhani et al. 2019 [25]	*	*	*	**		**	*		8
Yassouf et al. 2019 [26]			*	*		**	*		5
Sukhmani et al. 2019 [27]	*			*		**	*		6
Theppornpitak et al. 2021 [28]	*			**		**	*		6
Badur et al. 2021 [32]				*	**	**	*		6
Mahmoud et al. 2021 [29]			*	*	**	**	*		7
Chai et al. 2021 [30]	*	*	*	**	**	**	*		10
Lam et al. 2021 [31]	*			*	**	**	*		7
Cohort Studies									
Study	Representative of exposed cohort	Selection of non-exposed cohort	Ascertainment of exposure	Demonstration of outcome at start	Comparability	Assessment of outcome	Follow up for outcome	Follow up of cohorts	Score
Wolfe et al. 1985 [33]	*	*	*	*	**	*	*	*	9
Richardson et al. 1993 [37]	*	*	*		*	*	*	*	7
Mokhtar et al. 2013 [35]	*	*	*		*	*	*		6
Randomized Controlled Trial									
	Case definition	Representativeness of the cases	Selection of controls	Control definition	Comparability	Ascertainment of exposure	Same method ?	Non-response rate	Score
Elalfy et al. 2014 [36]	*	*		*	**	*	*	*	8

The Newcastle-Ottawa scale is a quality assessment tool that rates studies in three categories: research group selection, group comparability, and ascertainment of exposure or result of interest. The stars are considered as points provided to each quality item and serve as a quick visual assessment. A study with a NOS score of 7 or higher is rated high-quality; a study with 4 to 6 points ais deemed medium quality; and a study with 0 to 3 points are considered low quality [10]

### Compliance towards ICT

The included studies that reported the rate of ICT compliance are summarised in Table 3. Among these studies, 8 evaluated the compliance rate toward DFO monotherapy [17–21, 26, 33, 34], one compared DFO, DFP and DFX [35], and another compared DFO and DFX [22]. The compliance rates were compared between the groups of DFO & DFP and DFO & DFX in one study [36], while a different study compared DFO, DFP and DFX, and measured overall compliance [27]. Two studies [23, 29] measured but did not report the compliance rate while the remaining studies examined compliance toward ICT as a whole.

Generally, the rate of ICT compliance ranged from 20.93 to 75.3%. Specifically, the ICT compliance rate of the different agents ranged from 48.84 to 85.1% for DFO, 87.2–92.2% for DFP, and 90–100% for DFX. Based on the frequency of ICT administration, the rates of compliance ranged from 27.5 to 85.1%. There were a variety of ways to define compliance in the included studies with compliance defined as at least 4–7 days per week on DFO [17, 22], the number of DFO infusions > 50% of the calculated doses per month [29] or > 80% of the prescribed doses per year [18], percentage of the day in a month administering DFO > 90% [20], using the drugs at least 27 out of 36 months [24], and > 50 mg/kg/day of DFO or > 30 mg/kg/day of DFX [25]. The average compliance rates based on the vial or pill count were reported to range from 20.93 to 100% [19, 33–36]. In comparison, the average compliance rate was reported to be 54.9% in the study using MPR [26].

Meanwhile, six articles evaluated compliance using self-reported measurements. Among these studies, four reported a range between 75.3 and 91.4% of patients being compliant to ICT [27, 30–32] with the study by Theppornpitak et al. (2021) [28] reporting 22.9% of patients had high compliance levels. Haghpanah et al. (2013) [21] also reported that 85.1% of patients had good compliance levels although the authors did not mention how they defined compliance in the study.

### Studies’ findings based on outcomes

Overall, our review revealed a trend toward the advantages of ICT compliance in reducing serum ferritin, risk of cardiac and liver complications, and increasing patients’ HRQoL. For studies that reported these outcomes, the majority of the studies (N = 10/11, 90.9%) found a significant association between patients’ compliance and serum ferritin levels while most of the studies revealed that ICT compliance was linked to a lower risk of liver disease (N = 4/7, 57.14%) and cardiac disease (N = 6/8, 75%), endocrinologic morbidity (N = 4/5, 90%), and lower HRQoL (N = 4/6, 66.67%).

In total, 11 studies [17, 19, 20, 22, 26–28, 30, 33, 34, 36] examined the relationship between compliance rate and serum ferritin levels. Nine studies found a significant negative correlation between patients’ compliance, the average serum ferritin [17, 19, 22, 26, 27, 30, 34], and the mean decrease in serum ferritin prior enrolment to end of study [28, 33]. Concurrently, a study [36] showed better result (trend) in mean reduction in serum ferritin from baseline to the end of therapy in compliant group although there is no significance difference among the groups while the remaining one study [20] showed no significant relationship between compliance and serum ferritin levels despite most of the non-compliant patients having high serum ferritin levels (> 6000 µg/L). Furthermore, serum ferritin is proven as an important predictor of liver and cardiac iron load [25, 31], and endocrine complications [29, 31].

Seven studies in the review evaluated the relationship between the degree of ICT compliance and liver iron overload or complications. Compliance was shown to have a significant inverse association with liver iron overload or complication in 3 of the studies [27, 30, 34] Meanwhile, it showed the trend of higher compliance with higher mean decrease in mean liver iron concentration (LIC) level from baseline to end of treatment [36] while the remaining 3 studies revealed opposite results in liver iron load in MRI finding [25, 31] and prevalence of liver morbidities [35].

In addition, six studies revealed that compliance significantly reduces cardiac iron overload [25, 27], cardiac complications [19, 35], and the risk of developing the cardiac disease [33, 34] and a study revealed the better effectiveness in increasing the mean cardiac T2* value from the baseline [36]. In contrast, two articles reported no significant relationship between cardiac iron overload and compliance status [30, 31]. Mokhtar et al. (2013) [35] reported a significantly higher incidence rate of impaired left ventricular contractility in the non-compliant group (p = 0.021). The MRI finding reported in the study by Sukhmani et al. (2020) [27] revealed that non-compliant patients tend to have cardiac and severe hepatic overload, but there were no significant differences in the incidence of complications due to these findings between compliant and non-compliant groups.

Furthermore, endocrinologic complications or morbidities were shown to be significantly associated with poor ICT compliance (N = 4/5, 90%) [26, 27, 29, 31, 35] except in the study of Sukhmani et al. (2021) [27]. The endocrinologic morbidities in the studies including hypothyroidism, subclinical hypothyroidism, impaired glucose tolerance, impaired fasting glucose, diabetes mellitus, osteoporosis, and others. The number of non-compliant patients with hypothyroidism, overt and subclinical hypothyroidism were found significant higher [26] while the remaining studies examined the relationship using the number of patients with endocrinopathy (without specify) among the groups. The thyroid function tests such as thyroid stimulating hormone (TSH), free tri-iodothyronine (FT3), free thyroxine (FT4) and parathyroid hormone test (PHT) were used as parameter for the diagnosis of endocrine disorders in the studies [26, 27, 29], however, only the study by Yassouf et al. (2019) [26] evaluated the readings of TSH and FT4 among the groups and concluded non-compliance with DFO therapy raised the risk of thyroid dysfunction by 6.38 times. Meanwhile, the studies by Mokthar et al. (2013) [35] and Lam et al. (2021) [31] did not mention or evaluate the thyroid function.

In this review, only six studies evaluated the association between compliance and quality of life. The tools to measure HRQoL were Quality of Care (QoC) and Quality of Life (QoL) questionnaires [18]; Pediatric Quality of Life Inventory (PedsQL) [24]; Short Form-36 (SF-36) [21, 23, 36] and Transfusion-dependent QoL (TranQoL) [31] Four of the studies concluded the positive relationship between HRQoL and compliance, for example, patients with higher compliance had better HRQoL [18, 21, 24, 36]. However, the remaining two studies demonstrated no association between compliance and HRQOL for patients [23, 31].

**Table 3:** Chelation compliance measure and outcomes of published studies

Author, Year	Compliance Measure	Definition of Compliance	Compliance Rates	Outcomes
Wolfe et al. 1985 [33]	Vials count	Frequency of DFO administration (≥ 5 days per week)	Compliant: 47.22% (n = 17) Non-compliant: 52.78% (n = 19)	Compliant: reduced 𝛥 mean SF 1806 ± 760 ng/ml Non-compliant group: increased 𝛥 mean SF 1040 ± 234ng/ml (p < 0.05). Development of cardiac disease based on cardiac evaluation such as echocardiography & electrocardiography Compliant group: 5.88% (n = 1). Non-compliant group: 63.16% (n = 12).
Al-Refaie et al. 1992 [17]	Frequency of DFO administration.	Good compliance: DFO regularly for 4–5 nights weekly for 1 year.	Good compliance: 61.54% (n = 32) Poor compliance: 38.46% (n = 20)	Compliant: mean SF 1454 ± 1242 ng/ml. Non-compliant: mean SF 4686 ± 2866 ng/ml (p = 0.003). Compliant: NTBI values ranged − 1.5 to 6.0 µmol/l. Non-compliant: NTBI values ranged 2.1- 9.0µmol/l (p = 0.005).
Richardson et al. 1993 [34]	Vials count	Optimal compliance: >90%, Fair compliance: 50–90%, Poor compliance: <50% of prescribed DFO	Optimal compliance group: 60.53% (n = 46) Fair compliance group: 22.37% (n = 17) Poor compliance group: 17.10% (n = 13)	Compliance negatively proportional to SF with p < 0.001. Development of cardiac disease based on cardiac evaluation such as echocardiography & electrocardiography Optimal compliance: 30.43% (n = 14) Fair compliance: 83.35% (n = 14) Poor compliance: 69.23% (n = 9) Higher risk of developing cardiac disease was associated with fair compliance (p < 0.001) and poor compliance (p = 0.016). Compliance negatively proportional to liver iron (p < 0.001)
Arboretti et al. 2001 [18]	Percentage of DFO infusion over the year	Good compliance: >80%, fair compliance: 50–80%, poor compliance: <50% of insfusion per year.	good compliance group: 64% (n = 545) fair compliance group: 27% (n = 236) poor compliance group: 9% (n = 75) 11 missing data.	QoC questions: Good compliance group: 14% scored below 6 Fair/poor compliance group: 22% of good compliance group scored below 6 QoL questions: Good compliance group: 19% scored below 6 Fair/poor compliance group: 26% scored below 6.
Kidson-Gerber et al. 2008 [19]	Vials or pills count	The ratio of the amount dispensed to the prescribed dose over the year, classified into 0–24%, 25–49%, 50–74% and 75–100%.	Percentage DFO dispensed 75–100%: n = 9 Percentage DFO dispensed 50–74%: n = 12 Percentage DFO dispensed 25–49%: n = 8 Percentage DFO dispensed 0–24%: n = 14	Percentage DFO dispensed was inversely correlated with mean SF level with p < 0.001. Every 1% increase in DFO dispensed results in a reduction of 27 units in SF level. 28% of 0–24% DFO dispensed patients had high mean SF level which is more than 4000 ng/mL Inverse association between cardiac and/or endocrine complication with compliance with p = 0.02.
Lee et al. 2011 [20]	Percentage of days of DFO therapy over a month.	Highly compliant (> 90%), moderately compliant (51–90%), poorly compliant (0.1–50%) and not compliant (0%).	Highly compliant: 31% (n = 43), Moderately compliant: 50% (n = 70) Poorly or non-compliant: 19% (n = 26)	Patients correlated to SF level more than 6000ng/mL Compliant group: 38% (n = 14) Moderately compliant group: 38% (n = 25) Non-compliant group: 57% (n = 12) However, no significant relationship between patient self-reported compliance and their latest SF level with p = 0.186.
Haghpanah et al. 2013 [21]	N/A	N/A	Good compliance: 85.1% (n = 86) Poor compliance: 14.9% (n = 15)	SF-36 score (HRQoL) Good compliant group: 69.8 ± 14.6 Poor compliant group: 56.1 ± 19.5 with p = 0.002
Mokhtar et al. 2013 [35]	Vials or pills count	Good compliance: <50%, fair compliance: 50–80%, and poor compliance: >80% of the drug was returned.	DFO Non-compliance patients: 17.7% (n = 18) DFP Non-compliance patients 7.8% (n = 9) DFX Non-compliance patients: 0%	Based on clinical examination and echocardiography, non-compliant group had higher incidence of impaired left ventricular contractility with p = 0.021. The incidence of hepatic morbidities was unaffected by compliance. Non-compliance was associated with increased incidences of diabetes mellitus, hypogonadism, and mortality (p < 0.05, p < 0.05, p < 0.05)
Sobota et al. 2014 [23]	5-point Likert scale 1 = never, 2 = rarely, 3 = sometimes, 4 = often and 5 = a lot	Higher score indicated higher compliance.	N/A	Patient being transfused (general health domain only) and taking an oral chelator were related with higher HRQoL. For patients taking DFO alone, there was no correlation between any measure of compliance and HRQOL.
Bazi et al. 2017 [24]	Frequency of ICT administration	Regular chelation: at least 27 out of 36 months	Regular compliance: 27.5% (n = 22) Irregular chelation compliance: 71.3% (n = 57) No chelation: 1.2% (n = 1).	PedQL4 Regular compliance: PS: 56.66 Emo S: 75.00 SS: 25.68 ES: 51.33 Irregular compliance: PS: 55.76 Emo S: 66.84 SS: 26.93 ES: 52.68 QoL was inversely associated with patients on irregular chelation (p = 0.004). Overall, the total QoL score is 52.75 and 50.44 for regular and irregular chelation compliance.
Sobhani et al. 2019 [25]	Frequency of DFO administration or DFX consumption	Irregular users: <50 mg/kg/day (20 mg/kg/day in children) of DFO or < 30 mg/kg/day of DFX	Regular compliance: 67.8% (n = 61) Irregular compliance: 32.2% (n = 29)	Patients with higher SF had 2.068 folds more probability to have high liver iron load with p = 0.001 and 1.87 folds more probability to have high cardiac iron load with p = 0.001. Based on MRI T2*, patients with self-reported irregular use of iron chelating agents were more likely to have higher cardiac iron load. (p = 0.028) Based on MRI T2*, patients with irregular compliance was not significantly associated with liver iron load. (p = 0.110)
Yassouf et al. 2019 [26]	Medication possession ratio (MPR).	Compliant: MPR of at least 0.80.	Compliant group: 54.9% (n = 45) Non-compliant group: 45.1% (n = 37)	Mean SF level Compliant group: 3970.0 ± 1524.0 ng/mL Non-compliant group: 6953.0 ± 2690.0 ng/mL with significant difference p < 0.0001 TSH Compliant group: 2.45 ± 0.96 lIU/mL Non-compliant group: 4.38 ± 3.78 lIU/mL (p < 0.001) FT4 Compliant group: 1.25 ± 0.17 ng/dL Non-compliant group: 1.14 ± 0.22 ng/dL) (p < 0.005) 56.8% and 54.1% of DFO non-compliant patients having hypothyroidism and subclinical hypothyroidism with p < 0.0001 respectively It was found that non-compliance with DFO treatment elevates the incidence of thyroid dysfunction about 6.38 times when compared to DFO compliance.
Sukhmani et al. 2020 [27]	4 point Likert Scale	Compliant: >75% of the prescribed doses ( score 1 and 2), non-compliant: <75% (score 3 and 4)	Compliance score Compliant group 1: 26.5% (n = 57) 2: 62.8% (n = 135) Non-compliant group 3: 10.2% (n = 22) 4: 0.5% (n = 1) The compliance rate was highest with DFX (91.2%), followed by DFP (87.2%) and DFO (83.3%) (p = 0.350).	Mean SF level: compliant group: 2013.1 ± 1277.1 ng/mL Non-compliant group: 3129.8 ± 1573.2 ng/mL significantly lower with p = 0.000 Based on MRI T2*, cardiac iron overload were found higher in the non-compliant patients with p = 0.000 Based on MRI T2*, severe liver iron overload were found higher in the non-compliant patients with p = 0.021.
Theppornpitak et al. 2021 [28]	Thai version of the Morisky Medication Adherence Scales (MMAS-8)	Medium-low (> 1 score) and high groups (0 score).	High compliance level patient: 22.9% (n = 16) Medium-low compliance level patient: 77.1% (n = 54)	𝛥 mean SF 6 months prior to enrolment High compliance level: 276.4 ng/mL Medium-low compliance level: 413.0 ng/mL significant result with p = 0.034.
Badur et al. 2021 [32]	Frequency of using ICT	Never (did not use chelator), always (regular use of chelator) and sometimes (irregular use of chelator).	Did not use chelator: 11.1% (n = 3) regularly compliance: 55.6% (n = 15) irregularly compliance: 33.3% (n = 9)	There was no significant association among ICT compliance therapy and HRQoL with p = 0.552.
Mahmoud et al. 2021 [29]	Frequency of ICT administration	Good compliance: >50% of the calculated doses per month.	N/A	High serum ferritin levels were significantly associated with increased endocrine abnormalities with p = 0.003. Patients received 50% or less than 50% medication monthly tend to have endocrine disorder. (67.86% vs. 32.14%) p = 0.03. Increased endocrine abnormalities were significantly associated with poor ICT compliance p = 0.03.
Chai et al. 2021 [30]	Malay version of the Medication Compliance Questionnaire (MCQ).	Compliance:75% or higher.	Compliant group: 75.3% (n = 148) Non-compliant group: 24.7% (n = 50)	Significant association was observed between SF level and compliance status with p = 0.007. Amongst the non-compliant patients, 89.8% had serum ferritin level of ≥ 1000 mg/L compared with only 70.5% in patients who are compliant There was no significant relationship between cardiac MRI findings and compliance with p = 0.908. Liver MRI findings significantly associated with ICT compliance with p = 0.036. Patients who were non-compliant had 23.8% moderate liver abnormality and 61.9% severe liver abnormality, compared to 17.9% and 41.8% of compliant patients with moderate and severe liver abnormality.
Lam et al. 2021 [31]	Standardized questionnaire (not mentioned)	N/A	*>* 80% compliance: 63.8% (n = 37) 50–80% compliance 27.6% (n = 16) *<*50% compliance:8.6% (n = 5) 15 missing data	Cardiac iron loading was not significant associated with compliance. (p = 0.056) Liver iron loading was not significant associated with compliance. (p = 0.223) Endocrine complications were significantly associated with compliance. (p = 0.015) TranQOL is not significantly correlated to the compliance. (p = 0.352)

Abbreviations and Footnotes: DFO = desferrioxamine; DFP = deferiprone; DFX = deferasirox; ICT = iron chelation therapy; MRI = magnetic resonance imaging; N/A = not available; NTBI: Non-transferrin bound iron; PedsQL4 = Pediatric Quality of Life Inventory; PS = physical scale, Emo S = emotional scale; SS = social scale; ES = education scale; SF36 = Short Form-36; SF = serum ferritin; TranQOL = Transfusion-dependent QoL questionnaire; TSH = thyroid-stimulating hormone, QoL = quality of life; QoC = quality of care

## Discussion

Clinically, good compliance to chelation therapy has a great impact on disease control and the quality of life in thalassaemia patients. A high level of compliance is associated with significantly lower serum ferritin levels which tend to produce lower risk of iron overload complications, as well as a better quality of life. However, inadequate compliance to ICT therapy is common and patients are generally considered to have the lowest level of compliance to DFO and the highest level of compliance to DFX. Indeed, many studies measured the patients’ compliance or the clinical burden of thalassaemia itself, but not many studies measured the association among them. This systematic review identified and evaluated 20 medium to high quality articles that measured and compared the impact of chelation compliance on health outcomes or health-related quality of life. This would provide a clearer and a more comprehensive picture of the importance of compliance on various clinical outcomes for optimal management of thalassemia patients.

Almost all of the included studies (10 out of 11) that evaluated the association between compliance and serum ferritin levels reported significant negative correlations or trend among them, except the study by Lee et al. (2011) conducted in paediatric patients in Malaysia. This might be due to the markedly high average serum ferritin levels (6156 ± 4296 mg/L) of the patients in the study. Generally, iron chelator correlates better with a lower level of total body iron and leads to better therapy results [38]. However, it should be noted that the patients in the study by Lee et al. (2011) had only received DFO therapy for 2 years even though they had an average of 9 years of regular blood transfusions. A longer period of iron-chelation therapy will be needed to observe a significant decline in serum ferritin. This is further supported by the findings of Richardson and his colleagues which showed a prolonged administration of ICTs (early commencement) was associated with a greater reduction of serum ferritin [34].

Besides, the relationship between compliance levels and the risk of complications associated with ICT is still inconclusive. Several studies in our current review measured the risk of liver or cardiac iron overload complications through MRI [22, 25, 30] or cardiac evaluation such as clinical examination, echocardiography and, electrocardiography [33–35]. MRI T2* is an accurate and reliable tool to assess iron status in patients but it is sometimes not feasible due to high cost, and patients’ uncooperativeness to hold their breath throughout the process. Furthermore, MRI T2* is only applicable for patients aged 10 and above [4]. As a result, only a few articles reported the findings of MRIs involving a small sample size of patients explaining the unequivocal findings. Furthermore, previous literature showed that cardiac iron overload was discovered in cases of severe iron accumulation in the myocardium only [23]. Moreover, the age of the participants of these studies was relatively young. A large clinical study revealed that thalassaemia patients over 40 years old tend to develop and have a higher incidence of cardiac complications such as atrial fibrillation as well as a higher risk of stroke even without any evidence of iron overload [39]. This would suggest that further studies requiring a longer follow-up of participants are important to identify patients with iron overload complications.

Another interesting point to discuss regarding the association between medication compliance and HRQoL is that thalassaemia is a progressive disease. Symptoms of hyperferritinemia are often ambiguous and non-specific, and commonly present without causing any early real clinical manifestations. Hence, a high level of serum ferritin may not affect thalassaemia patients in their daily life, but the accumulation of excess iron may result in the occurrence of life-threatening complications in later life. Some thalassaemia patients receiving DFO therapy have lower chelation compliance due to the inconvenient administration procedure and pain at the injection site. Avoiding such an inconvenient procedure may temporarily improve their current quality of life, however, the long-term consequences of increased iron loading will be more likely to result in reduced quality of life in the future [40]. Additionally, generalising and comparing patients’ health-related quality among these studies is challenging due to thevariations in the tools used for measuring HRQoL.

Anyway, the objective of our systematic review is very similar to the review by Delea et al. in 2007 [10]. In terms of compliance rate, Delea et al. found a slightly lower range of mean scores towards DFO (59-78%) and DFP (79-98%) in comparison to our study. However, they were unable to include any studies that looked into DFX (not available during the conduct of the review by Delea et al. 2007). Although Delea et al. systematically reviewed 18 studies on compliance rates with ICT, they only managed to include 5 articles to discuss the association between compliance and the incidence of iron overload complications. The review demonstrated a higher incidence of cardiac and endocrinopathies complications in poor compliant patients. The five articles included in the previous review represented studies conducted in Western and developed countries only (the United States, Australia, and Italy) and all were performed before 2000, making it difficult to generalise the findings for developing countries such as Malaysia. Moreover, after two decades, the clinical management of thalassaemia population has improved due to more options of ICT and improved healthcare technologies in recent years. Our review also included two articles from the previous review since they fulfilled our inclusion criteria. As a result, our review provided updated information on the impact of compliance on thalassaemia patients.

Based on the findings from our study, several recommendations for future studies could be suggested in regard to the impact of chelation compliance on iron overload complications. In the included studies in our current review, the sample sizes used to assess the relationship between compliance and iron overload complications were relatively small, ranging from 36 to 90, except for the study by Mokhtar et al. (2013) which had a sufficient sample size (n = 447). For any study, an adequate sample size always allows statistically more reliable conclusion to be derived from the results. Secondly, researchers should consider and recruit patients of older age in the study as higher risk and incidence of iron overload complications were found in older thalassaemia patients (> 40 years old) [39]. In the studies included in our review, the median age of the samples ranged from 11.34 to 22.7 years old. Researchers may also design studies with different age groups and compare the impact of poor compliance. Besides, to appropriately identify patients with incidence of iron overload complications, a longer period of study is needed.

Lastly, our review does contain some limitations. Multiple confounding factors, including the age and sex of patients, as well as the frequency of blood transfusions, may contribute to variations in the level of patient’s compliance. The evaluation of these factors is beyond the scope of our current review due to the lack of data in the included studies. We also did not include non-English language studies in this review. In addition, grey literature was also not included due to its diversity and challenges to assess the quality of the literature. The exclusion of these articles may cause the exclusion of potentially valuable data. However, the available evidence in our review is considerably more appropriate and robust to fill the gaps in the topic.

To our best knowledge, this is the first review with worldwide data from developed and developing countries that demonstrated the positive impact of compliance in improving health outcomes (especially serum ferritin, cardiac, liver, and endocrinologic complications), and patients’ HRQoL. The conclusion of the current review was drawn and supported by more than 50% of the studies reviewed. Nevertheless, we were unable to perform a meta-analysis to provide a more precise estimate of the association between compliance and the various outcomes due to the limited number and heterogeneity of studies reporting the different outcomes of interest.

## Conclusion

Congruent with expectation, our review demonstrated that compliance to iron chelators maximises the benefits of the therapy in reducing serum ferritin, iron overload complications and HRQoL. The serum ferritin levels appear to be the most affected outcome by compliance, while the relationship between ICT compliance, iron overload complications, and HRQoL was shown but further investigation is needed. To fully understand the impact of compliance on the most vulnerable patient groups, more comprehensive research with larger sample size and comparing the impact of ICT to the outcomes of interest among different age groups is needed.

## Data Availability

Not applicable.
